# Short-Term Prognostic Efficacy of mGPS and LCS in Patients With Acute Heart Failure

**DOI:** 10.3389/fcvm.2022.944424

**Published:** 2022-07-05

**Authors:** Jing Wang, Ling Xie, Ping Lyu, Feng Zhou, Hong-Li Cai, Rong-Xing Qi, Qing Zhang

**Affiliations:** ^1^Department of Cardiology, Affiliated Hospital 2 of Nantong University, Nantong, China; ^2^Department of Clinical Laboratory, Affiliated Hospital 2 of Nantong University, Nantong, China; ^3^Department of General Practice, Affiliated Hospital 2 of Nantong University, Nantong, China; ^4^Department of Image, Affiliated Hospital 2 of Nantong University, Nantong, China

**Keywords:** acute heart failure, inflammation, LCS, mGPS, prognosis

## Abstract

**Aim:**

Systemic inflammation plays an important role in the occurrence and development of acute heart failure. The modified Glasgow Prognostic Score (mGPS) and “lymphocyte C-reactive protein score” (LCS) are used to assess the inflammation levels in cancer patients. The purpose of this study was to assess the prognostic value of these two inflammation-related scoring systems in patients with acute heart failure.

**Methods:**

Two hundred and fifty patients with acute heart failure were enrolled in this study. The mGPS and LCS scores were recorded after admission. All patients were divided into 2 groups: the death group and the survival group according to the 3-month follow-up results. The predictive values of mGPS and LCS were assessed using receiver-operating characteristic (ROC) analyses. Univariate and multivariate logistic analyses were used to evaluate the relationships between variables and endpoint.

**Results:**

The levels of mGPS and LCS in the death group were significantly higher than those in the survival group (P < 0.05). The areas under the ROC curve of the mGPS and LCS for predicting death were 0.695 (95%CI: 0.567~0.823) and 0.736 (95%CI: 0.616~0.856), respectively. Multivariate analysis demonstrated that both LCS, LVEF and serum direct bilirubin were independent predictors of all-cause death, excluding mGPS.

**Conclusions:**

Compared with mGPS, LCS is independently associated with short-term outcomes in patients with acute heart failure. LCS was a clinically promising and feasible prognostic scoring system for patients with acute heart failure.

## Introduction

The incidence of acute heart failure (AHF) has been increasing year by year, and has become the most common reason for unplanned admission of patients over 65 years old (1). Patients with acute heart failure have high hospital costs and long-term mortality. Prognostic assessment has played an increasingly important role in the treatment of these patients. The inseparable association of inflammation and heart failure has long been recognized in many studies (2, 3). Recently, albumin (4, 5), C-reactive protein (6, 7) and lymphocytes (8, 9) have also been reported to predict the prognoses of patients with ADHF, sometimes even better than brain natriuretic peptide in predicting in-hospital death (10).

The modified Glasgow prognostic score (mGPS) and lymphocyte C-reactive protein score (LCS), these two new and readily available markers of inflammation, were initially shown to have independent prognostic value in cancer patients (11–13). Many studies have focused on the correlation between mGPS and prognosis of cardiovascular diseases (14–16), and it has been proved to be an effective and reliable prognostic indicator for patients with acute and chronic heart failure. Recently, Okugawa et al. developed a new inflammatory scoring system based on lymphocyte count and serum CRP level, known as the LCS (12), which has not been demonstrated the effect on prognosis in patients with AHF.

The aim of this study was to evaluate the clinically prognostic efficacy of mGPS and LCS for mortality in patients with AHF and to determine the most clinical practical scoring system for predicting outcomes of patients with AHF.

## Methods

### Study Population

A total of 250 patients with AHF who were hospitalized in the Department of Cardiology, The Second Affiliated Hospital of Nantong University from October 2019 to October 2020 were eventually included in this study (156 males and 94 females). All patients met the diagnostic criteria for acute heart failure (17).

Patients with rheumatic disease; patients with hematological disease; patients with malignant tumors; patients with chronic or infectious disease; patients with incomplete data and lost to follow-up and pregnant patients were excluded from the study. Patients were also excluded due to chronic or infectious disease or due to taking immunosuppressive drugs for disease control that may influence the status of mGPS and LCS.

Two hundred and fifty-eight patients were initially enrolled in this study, but 250 patients were eventually included according to the exclusion criteria. Of those, 5 patients were excluded because they lacked one of the lymphocyte, CRP and albumin data. For these 5 patients, follow-up was still carried out, and no death occurred in 5 patients. In addition, only 3 patients were lost to follow-up in this study, and these patients were also excluded.

The investigation conformed to the principles outlined in the Declaration of Helsinki. The study was approved by the Ethics Committee of The Affiliated Hospital 2 of Nantong University, Nantong (IRB number: 2019KN104), and informed consent was obtained from all patients.

### Data Collection

Demographic data (age, gender, body mass index (BMI), blood pressure, heart rate and treatment) was obtained from medical records. Diagnoses of hypertension and diabetes mellitus, and dyslipidemia were obtained from the patients' medical records or records of patient histories of previous medical therapy. The levels of lymphocyte count, leucocytes, C-reactive protein (CRP) and N-terminal brain natriuretic peptide (NT-proBNP) were measured immediately after admission. All the other measurements such as aspartate transaminase (AST), albumin, total bilirubin, direct bilirubin, total cholesterol, serum creatinine and urea nitrogen were performed on the second day of hospitalization, after fasting. Echocardiographic parameters include left ventricular ejection fraction (LVEF), left atrial diameter (LAD), left ventricular end diastolic diameter (LVEDD), and left ventricular end systolic diameter (LVESD). The glomerular filtration rate was estimated using the chronic kidney disease epidemiology collaboration (CKD-EPI) equation (18). Body mass index (BMI) was calculated as kg/ m^2^.

### Follow-Up

All patients were prospectively followed up for 3 months or until death. All-cause mortality was defined as the study end point, and data was obtained from the telephone interview and outpatient visits.

### The Definition of Two Scores

The mGPS, defined based on the combination of serum CRP and albumin, was described as Table 1. Patients with both CRP ≤ 10 mg/L and albumin ≥35 g/L were classified to a score of 0; patients with either CRP >10 mg/L or albumin ≥35 g/L were classified to a score of 1 and patients with both CRP >10 mg/L and albumin <35 g/L were classified to a score of 2 (12).

**Table 1:** Two types of systemic inflammation-based prognostic scores.

**LCS**	**Points allocated**
Lym ≥ 1 ×10^9^/L and CRP ≤ 3 mg/L	0
Lym <1 ×10^9^/L and CRP <3 mg/L	1
Lym > 1 ×10^9^/L and CRP > 3 mg/L	1
Lym <1 ×10^9^/L and CRP > 3 mg/L	2
**mGPS**	
CRP ≤ 10 mg/L and albumin ≥ 35 g/L	0
CRP > 10 mg/L and albumin ≥ 35 g/L	1
CRP ≤ 10 mg/L and albumin <35 g/L	1
CRP > 10 mg/L and albumin <35 g/L	2

*CRP, C-Reactive protein; LCS, lymphocyte C-reactive protein score; Lym, Lymphocyte count; mGPS, modified Glasgow prognostic score*.

The LCS, was established using the circulating lymphocyte count and CRP level as mentioned earlier. Patients with both the lymphocyte count ≥ 1 × 10^9^/L and CRP ≤ 3 mg/L were scored as 0. If only one parameter changes, that is, lymphocyte count <1 × 10^9^/L or CRP >3 mg/L, 1 point was allocated. If both parameters were altered, that is, CRP>3 mg/L and lymphocyte count <1 × 10^9^/L, patients received 2 points (Table 1) (11).

### Statistical Analysis

Baseline continuous variables were presented as mean ± standard deviations (SD) or median with the first and fourth quartile (Q1–Q4); depending on the distribution of the data. Categorical data is presented as counts and percentages. For comparisons between the patient groups with different endpoints, the independent samples *t*-test, the Mann–Whitney *U* test, and the χ^2^ test were used. Multivariate analysis using stepwise logistic regression model tested variables that were significant (*P* < 0.1) in the univariate analysis to determine independent predictors of all-cause mortality. Receiver operating characteristic curve (ROC) was used to evaluate the predictive value of various independent predictors for mortality in patients with acute heart failure. For all tests, a *p*-value < 0.05 was considered statistically significant. Analyses were performed with the statistical package SPSS 25.0 (SPSS Inc., Chicago, IL).

## Results

### Patient Characteristics

Two hundred and fifteen patients were included in the study. Demographic, clinical, and laboratory characteristics of patients on admission who reached the endpoint are shown in Table 2. The all-cause mortality rate was 6% in 3 months. The levels of LCS and mGPS in the death group were significantly higher than those in the survival group (*P* < 0.05). Besides this, the death group had lower LVEF, systolic and diastolic blood pressure, but higher CRP, AST, creatinine, urea nitrogen, NT-proBNP levels, LVESD and incidence of previous myocardial infarction events than the survival group (*P* < 0.05). No significant differences were observed with respect to gender, age, BMI, white blood cell count, lymphocyte count, hemoglobin, total bilirubin, direct bilirubin, total cholesterol, eGFR and treatment.

**Table 2:** Characteristics of patients who reached and did not reach the primary outcome.

**Variables**	**Totality** **(*n* = 250)**	**Survivors** **(*n* = 235)**	**Non-survivors** **(*n* = 15)**	***p*-value**
Gender, male%	156 (62.4)	144 (61.3)	12 (80)	0.147
Age, years	75 (65, 80)	74 (65, 80)	79 (57, 81)	0.479
BMI, kg/ m^2^	23.56 (20.81, 26)	23.50 (20.81, 25.92)	22.89 (20.22, 27.16)	0.927
Systolic pressure, mmHg	125 (109, 138.25)	126 (110, 139)	103 (91, 125)	**0.023**
Diastolic pressure, mmHg	76 (65, 87)	76 (67, 87)	62 (56, 80)	**0.018**
Heart rate	85 (71, 100.25)	85 (71, 100)	84 (66, 110)	0.825
Smoking	53 (21.20)	49 (20.85)	4 (26.67)	0.593
NYHA IV	119 (47.6)	107 (45.5)	12 (80.0)	**0.010**
Comorbidities				
Hypertension	134 (53.60)	127 (54.04)	7 (46.67)	0.579
Diabetes	65 (26.00)	61 (25.96)	4 (26.67)	0.952
Atrial fibrillation	117 (46.80)	111 (47.23)	6 (40)	0.585
Prior MI	39 (15.60)	33 (14.04)	6 (40)	**0.007**
Laboratory data				
Leucocytes, 10^9^/L	9.0 (6.9, 11.2)	6.9 (5.1, 9.1)	7.0 (3.8, 8.1)	0.601
Lymphocyte count, 10^9^/L	1.1 (0.8, 1.5)	1.1 (0.8, 1.6)	0.9 (0.7, 1.3)	0.106
Hemoglobin, g/L	124 (113, 135.25)	124 (113, 135)	122 (112, 137)	0.760
CRP, mg/L	7.70 (2.55, 20.89)	6.76 (2.17, 20.06)	40.08 (10.89, 92.91)	**0.001**
Albumin, g/l	36.10 ± 4.09	36.21 ± 4.08	34.34 ± 3.96	0.087
AST, U/L	29 (21, 54.75)	28 (20, 53)	49 (26, 90)	**0.030**
Total bilirubin, μmol/L	15.75 (10.9, 24.08)	15.6 (10.85, 23.7)	18.7 (10.9, 49.7)	0.200
Direct bilirubin, μmol/L	5.4 (3.5, 9.0)	5.3 (3.5, 8.9)	8.4 (4.8, 22.5)	0.086
Total cholesterol, mmol/L	3.72 (2.97, 4.41)	3.75 (2.98, 4.42)	3.46 (2.32, 4.00)	0.099
Creatinine, μmol/L	85.25 (70, 108.38)	85 (69.80, 105.95)	114 (82, 144)	**0.018**
Urea nitrogen, mmol/L	7.31 (5.30, 9.84)	7.22 (5.26, 9.71)	9.07 (7.07, 21.20)	**0.020**
eGFR, ml/min	70.64 (50.08, 90.08)	71.6 (50.23, 90.89)	56.22 (39.23, 80.9)	0.063
NT-proBNP, pg/ml	5,575 (2824.5, 11,062)	5,255 (2753.25, 10,855)	8,365 (5,762, 20,115)	**0.014**
Echocardiographic data				
LVEF, %	46 (35, 59)	46 (36.3, 60)	30.5 (24.5, 46)	**0.003**
LVESD, mm	43 (34, 52.5)	42.5 (34, 52)	50 (43.5, 65.5)	**0.021**
LVEDD, mm	57 (50, 65)	57 (49.25, 65)	61 (55.5, 73.5)	0.052
Treatment				
ACEI/ARB	158 (63.2)	151 (64.3)	7 (46.7)	0.179
βblocker	179 (71.6)	170 (72.3)	9 (60.0)	0.375
Diuretics	231 (92.4)	219 (93.2)	12 (80.0)	0.094
LCS				
0	40 (16)	40 (17.02)	0	
1	141 (56.4)	136 (57.87)	5 (33.3)	**0.001**
2	69 (27.6)	59 (25.11)	10 (66.67)	
mGPS				
0	107 (42.8)	105 (44.7)	2 (13.3)	
1	87 (34.8)	81 (34.5)	6 (40)	**0.006**
2	56 (22.4)	49 (20.9)	7 (46.7)	

*AST, aspartate transaminase; BMI, body mass index; eGFR, estimated glomerular filtration rate; LVEF, left ventricular ejection fraction; LVEDD, left ventricular end diastolic diameter; LVESD, left ventricular end systolic diameter; MI, myocardial infarction; NYHA, New York Heart Association; NT-proBNP, N-terminal pro B-type natriuretic peptide*.

*Values with P < 0.05 in the table are shown in bold*.

### Predicting Clinical Outcome

On univariate analyses, higher New York Heart Association class, CRP, total bilirubin, direct bilirubin, urea nitrogen, LVESD, LCS and mGPS but lower LVEF, systolic and diastolic blood pressure at admission were significantly associated with outcome (*P* < 0.05; Table 2). Variables that had a *p*-value < 0.1 in the univariate analyses were used in a multivariate logistic regression analysis model. After adjusting for other potential confounding factors, multivariate analyses showed that only direct bilirubin (OR: 1.096, 95%CI: 1.019~1.178, *p* = 0.014), CRP (OR: 1.036, 95%CI: 1.008~1.065, *p* = 0.011), LVEF (OR: 0.845, 95%CI: 0.750~0.952, *p* < 0.01) and LCS (OR: 11.694, 95%CI: 1.433~95.409, *p* = 0.022) at admission were independently associated with the mortality outcomes (Table 3).

**Table 3:** Univariate and multivariate analysis of variables associated with prognosis in patients with acute heart failure.

**Variable**	**Unadjusted OR (95% CI)**	***P*-value**	**Adjusted OR (95% CI)**	***p*-value**
NYHA	4.785 (0.057–0.760)	0.017		
Systolic pressure	0.979 (0.958–1.000)	0.049		
Diastolic pressure	0.956 (0.918–0.994)	0.024		
Albumin	0.900 (0.796–1.016)	0.088		
Total cholesterol	0.607 (0.341–1.082)	0.091		
Total bilirubin	1.032 (1.006–1.058)	0.016		
Direct bilirubin	1.057 (1.013, 1.104)	0.012	1.096 (1.019–1.178)	0.014
Urea nitrogen	1.093 (1.028–1.162)	0.004		
eGFR	0.983 (0.963–1.003)	0.087		
CRP	1.016 (1.007–1.026)	0.001	1.036 (1.008–1.065)	0.011
LVEF	0.942 (0.902–0.983)	0.006	0.845 (0.750–0.952)	0.006
LVESD	1.052 (1.008–1.098)	0.021		
LVEDD	1.048 (0.998–1.101)	0.058		
LCS	5.286 (1.876–14.896)	0.002	11.694 (1.433–95.409)	0.022
mGPS	2.541 (1.256–5.141)	0.009		

### ROC Curve Analysis

**Figure 1:**
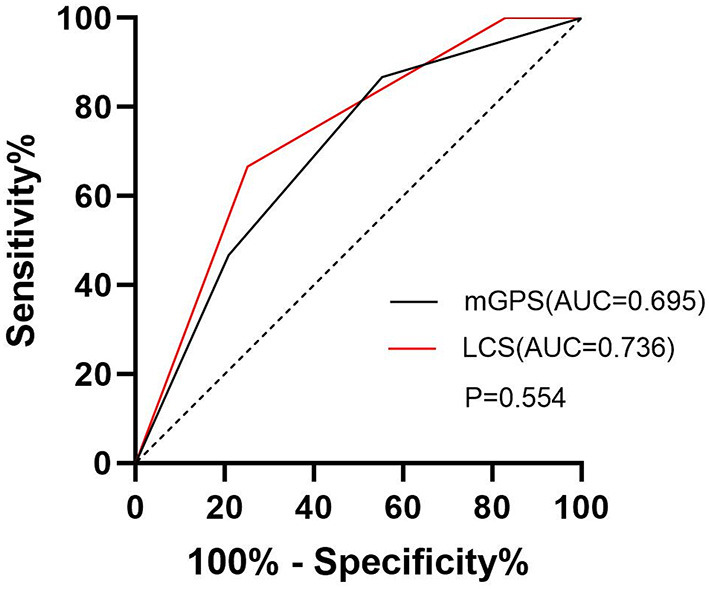
Receiver operating characteristic curves for LCS, and mGPS in the prediction of mortality in patients with acute heart failure. The area under the receiver operating characteristic curve (AUC) for LCS was 0.736, AUC for mGPS was 0.695.

The area under the ROC curve of the mGPS for predicting death was 0.695, with a cut-off level of 0.5 (95%CI: 0.567~0.823), sensitivity of 86.7%, and specificity of 44.7% (*p* < 0.01; Figure 1). The area under the ROC curve of the LCS for predicting death was 0.736, with a cut-off level of 1.5 (95%CI: 0.616~0.856), sensitivity of 66.7%, and specificity of 74.9% (*p* < 0.01; Figure 1). Although the AUC of LCS was slightly larger, there was no statistical difference between these two methods (*P* > 0.05; Figure 1). However, the results of multivariate analyses showed that only LCS was independently associated with the prognosis of patients with acute heart failure.

## Discussion

In recent years, more and more studies have elucidated the key role of inflammatory biomarkers in the development of acute heart failure (7, 19–21). Although the role of inflammation in heart failure has been recognized, inflammation scores have not been included in the risk assessment of acute heart failure, and this gap needs to be filled. As mGPS, an inflammatory-based prognostic risk score, has been gradually explored in predicting the prognosis of patients with different types of cancer, cardiovascular physicians are discovering its value in predicting the poor prognosis of patients with acute and chronic heart failure (14–16). In 2019, LCS developed by Okugawa et al. was also proved to be a reliable marker of inflammatory response in patients with gastric cancer (12). To our knowledge, this is the first study to evaluate the predictive value of LCS in patients with acute heart failure and the first study to compare the correlation between mGPS, LCS and prognosis in patients with acute heart failure.

The study showed that the levels of LCS and mGPS in the death group were significantly higher than those in the survival group. Although both mGPS and LCS were associated with 3-month mortality in the univariate analyses (*p* < 0.05), and there was no significant statistical difference of the AUC between these two scoring systems, only LCS is found to be independently associated with prognosis after adjustment for relevant factors, while mGPS was excluded. In conclusion, LCS has a more promising application in predicting the prognosis of acute heart failure.

The increased level of tumor necrosis factor-α (TNF-α) in the circulation of patients with chronic heart failure since Levine (22) first advocated it in 1990, has been widely explored for decades, and accumulating studies have elucidated the pivotal role of the inflammatory biomarkers in acute and chronic heart failure (7, 19–21). The inflammation-based prognostic score LCS combining CRP and lymphocyte count was originally used to measure systemic inflammatory status and predict prognosis in cancer patients. Similar to heart failure, cancer is also a systemic disease with activated inflammatory response. Previous studies have shown that low lymphocyte counts can help identify patients at higher risk of death in heart transplant patients and in patients with various types of acute or chronic heart failure (9, 23). In addition, high CRP levels on admission and discharge are considered to be closely associated with poor prognosis in patients with acute decompensated heart failure (7, 24). In the LCS scoring system, a lower lymphocyte count and a higher CRP level are assigned a higher score. In fact, several previous studies have hinted in part at the predictive power of LCS in patients with acute heart failure. The elevation of these inflammatory biomarkers in ADHF indicates that ADHF patients are in a significant systemic inflammatory state. In the LCS scoring system, a lower lymphocyte count and a higher CRP level are assigned a higher score, quantifying the inflammatory status and providing a more comprehensive measure of systemic inflammation. This study confirmed the relationship between a more activated inflammatory state and worse prognosis in acute heart failure and reported the successful implementation of the cancer-cohort-derived LCS risk score to a cohort with AHF patients.

## Conclusions

In conclusion, this study has shown the clinical utility of the simple and objective inflammation-based score in acute heart failure patients. An activated inflammatory state appears to be characteristic for a more advanced disease. Compared with mGPS, LCS is independently associated with short-term outcomes in patients with acute heart failure. The LCS may help clinicians to identify AHF patients with worse prognosis, for whom more intensive and aggressive treatment may be needed and thus improve their prognosis.

## Limitations

This study was retrospective and all patients enrolled were from the same institution. In the next step, we will include a larger sample size to validate the conclusions of this study and determine whether controlling inflammatory levels in patients with acute heart failure improves patient outcomes. Besides, the follow-up time of this study was only 3 months, so the follow-up time could be extended for further verification.

## Data Availability Statement

The original contributions presented in the study are included in the article/supplementary material, further inquiries can be directed to the corresponding authors.

## Ethics Statement

The studies involving human participants were reviewed and approved by the Ethics Committee of The Affiliated Hospital 2 of Nantong University, Nantong (IRB number: 2019KN104). The patients/participants provided their written informed consent to participate in this study.

## Author Contributions

JW: conceptualization, methodology, investigation, data curation, formal analysis, writing—original draft, and writing—review and editing. LX: conceptualization, methodology, investigation, data curation, and writing—original draft. PL, FZ, and H-LC: investigation and data curation. R-XQ: resources, writing—review and editing, visualization, project administration, and supervision. QZ: resources, formal analysis, writing—original draft, writing—review and editing, visualization, project administration, and supervision. All authors contributed to the article and approved the submitted version.

## Funding

This work was supported by the Scientific Research Project of Nantong Municipal Health Commission (MA2020004, MB2021010), Project of Nantong Science and Technology Bureau (JCZ21099), and Kangda College of Nanjing Medical University (KD2021KYJJZD012).

## Conflict of Interest

The authors declare that the research was conducted in the absence of any commercial or financial relationships that could be construed as a potential conflict of interest.

## Publisher's Note

All claims expressed in this article are solely those of the authors and do not necessarily represent those of their affiliated organizations, or those of the publisher, the editors and the reviewers. Any product that may be evaluated in this article, or claim that may be made by its manufacturer, is not guaranteed or endorsed by the publisher.

## Abbreviations

AHF: acute heart failure
mGPS: modified Glasgow prognostic score
LCS: lymphocyte C-reactive protein score
BMI: body mass index
CRP: C-reactive protein
NT-proBNP: N-terminal brain natriuretic peptide
AST: aspartate transaminase
LVEF: left ventricular ejection fraction
LAD: left atrial diameter
LVEDD: left ventricular end diastolic diameter
LVESD: left ventricular end systolic diameter
CKD-EPI: chronic kidney disease epidemiology collaboration
TNF-α: tumor necrosis factor-α.
